# Medial migration of the tympanostomy tube: what is the optimal management option?

**DOI:** 10.11604/pamj.2019.34.216.19472

**Published:** 2019-12-30

**Authors:** Ilias Benchafai, Mohamed Moumni, Saloua Ouraini, Noureddine Errami, Bouchaib Hemmaoui, Fouad Benariba

**Affiliations:** 1 Department of Otolaryngology-Head and Neck Surgery, Mohamed V Military Hospital, Rabat, Morocco

**Keywords:** Medial migration, tympanostomy tube, etiology, optimal treatment

## Abstract

The tympanostomy tube insertion is the gold standard of treatment for secretory otitis media. Complications are associated with this surgery in 17% of cases. One of the rare but real complications is the medial migration of the tympanostomy tube. To our knowledge, this is the 14^th^ case reported in childhood. Considering this rarity, there is no consensus for the management of this phenomenon. Some authors propose a surgical removal, while others prefer to observe whether the patient is asymptomatic. We reported a case of migration of the tympanostomy tube and described the detailed clinical features and management options. This case report and mini-review will broaden readers the knowledge of medial migration of the tympanostomy tube and may guide the relative treatment of this complication in the future.

## Introduction

Secretory otitis media is one of the most common otological pathology-affecting children, between 3 to 7 years of age [1]. Since reintroducing tympanostomy tube insertion for the treatment of otitis media with effusion [1], it has constantly been one of the commonest surgeries performed in children under general anesthesia [2]. It is estimated that tympanostomy tube placement is associated with some sort of complications in 17% of cases [3, 4]. The commonest of these complications include otorrhea, scarring granulation tissue, tympanosclerosis, blockage of the tube lumen, premature extrusion and permanent perforation. Cholesteatoma is a rare but more serious complication. An additional rare complication is the medial migration of the tympanostomy tube into the middle ear space, as opposed to normal extrusion into the external auditory canal. This complication has been reported to occur in 0 - 1.1% of patients [5]. Although not fully understood, the underlying mechanisms behind this complication are believed to be: an oversized myringotomy, a eustachian tube dysfunction or a technical error in insertion [4, 6]. To the best of our knowledge, only 14 cases of medial migration of the tympanostomy tube in children were reported in the literature [7]. Given this rarity, it is understandable why there is no consensus dedicated to the optimal management of this condition. The aim of this report is to use an individual case of medial tube migration, to better define the clinical features of this entity, theorize possible etiologies and discuss treatment recommendations, in order to delineate the most appropriate management option.

## Patient and observation

**Figure 1:**
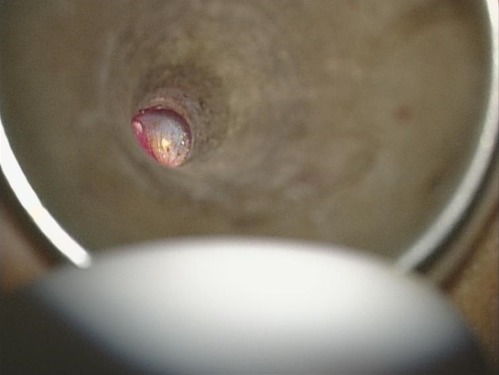
Otoscopy showing a blue shadow of the grommet through the left ear drum

**Figure 2:**
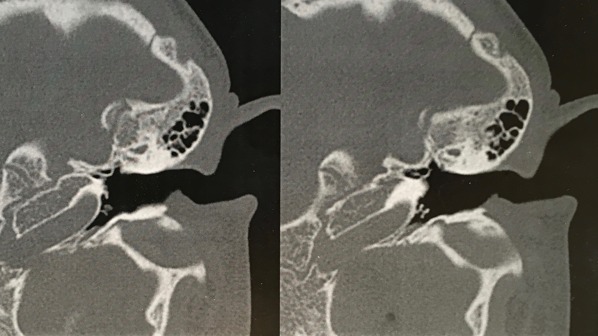
CT showing the presence of a tympanostomy tube in the left middle ear space

**Figure 3:**
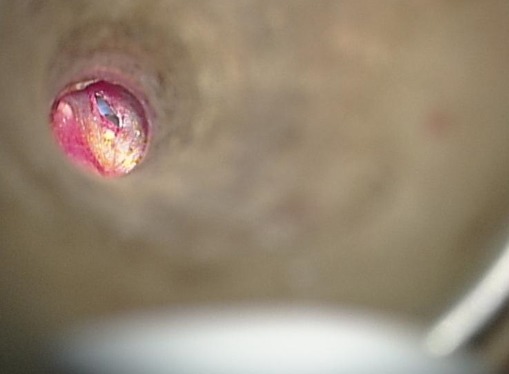
Per-operative view showing the presence of the tympanostomy tube in the middle ear cleft through a myringotomy incision

**Figure 4:**
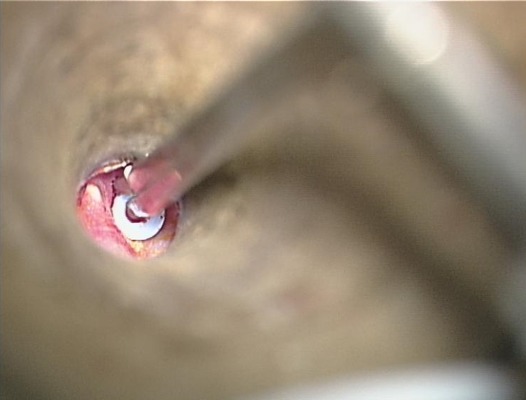
Per-operative view showing extraction of the tympanostomy tube

A 10-year-old boy had a history of bilateral tympanostomy tubes insertion one year ago. Both tympanostomy tubes were seen in place in the eardrum during the first two post-operative visits at two months and six months after the operation. The child later developed left hearing loss and ear fullness sensation. During his 12^th^ month visit, both tympanostomy tubes were noted to be missing. The microscopic examination of the ear was carried out and a blue shadow of the grommet was seen through the left eardrum (Figure 1). The right tympanic membrane was noted to be intact, and it was presumed that the right tube had extruded. Audiometric evaluation done at that time demonstrated mild conductive deafness in the left ear. The right ear had a normal hearing. The computed tomography (CT) of the temporal bone showed a foreign body in the left middle ear space attesting to the presence of the tympanostomy tube (Figure 2). Surgical exploration under general anesthesia via the external auditory canal making an incision in the anteroinferior quadrant allowed extraction of the tympanostomy tube (Figure 3, Figure 4).

## Discussion

Tympanostomy tube insertion is one of the commonest operations performed in Morocco. In 17% of cases, this procedure is complicated by otorrhoea, tympanosclerosis, residual perforation, granulation tissue formation and cholesteatoma [3, 4]. Medial migration of tympanostomy tube is rare but real complication, it occurs in 0,5% of cases [5]. While common complications have been readily studied, there have been very few studies dedicated to the medial migration of the tympanostomy tube. This rare occurrence where the tube migrates into the middle ear space as opposed to normal extrusion laterally into the external auditory canal. Clinically, medial migration of tympanostomy tube is either asymptomatic discovered accidentally during a control visit, or symptomatic and it's manifested by a sensation of ear fullness, permanent otorrhea through a persistent perforation, also by a transmission deafness due to middle ear effusion or ossicular lysis, more rarely by cholesteatoma and exceptionally by perilymphatic fistula [8]. Prior case reports noted Amstrong, Shah and Shepard tubes, this suggests that medial displacement is independent of tube type [9] and occurs at different intervals after placement (Table 1). The mechanism behind this phenomenon is not completely understood. The authors proposed several theories. One of these hypotheses is an abnormally long myringotomy incision. They assure that with an oversized incision the outer rim of the tube is lying partially inside the eardrum when inserted, preventing keratin from bringing accumulated in its groove; the normal mechanism for extrusion of tympanostomy tube [4, 9, 10]. Also, a recurrent ear infection in the post-operative period could change a myringotomy into a perforation. Consequently, this may result in a loosely fitting tube slipping into the middle ear [9, 10].

**Table 1:** List of cases of tympanostomy tube migration in children published in the literature

Author	Number of patients	Age in years	Type of tubes	Timing of migration	Symptoms	Treatment
Groblewski *et al.*	5	3	Amstrong	0.75 month	Asymptomatic	Removal
		3	Donaldson	16 months	Speech delay	Removal
		5	Pope	NA	Speech delay	Removal
		5	Paparella	32 months	Speech delay	Removal
		7	Amstrong	14 months	Asymptomatic	Removal
Roy *et al.*	3	8	NA	NA	Hearing loss	Removal
		6	NA	NA	Hearing loss	Removal
		3	NA	16 months	Eardrum perforation	Removal
Kumar *et al.*	2	6	Shah	30 months	NA	Removal
		11	Shah	84 months	Eardrum perforation	Removal
Green *et al.*	2	12	NA	48 months	Asymptomatic	Observation
		12	NA	12 months	Asymptomatic	Observation
Cunninghan *et al.*	1	9	NA	24 months	NA	Removal
Our case	1	10	Shepard	12 months	Ear fullness Hearing loss	Removal

Another theorized mechanism is that medial displacement of the tympanostomy tube may occur as a result of persistent negative middle ear pressure due to eustachian tube abnormalities. If strong enough, this negative pressure can counteract the force of epithelial migration, even more, if the tube lumen is blocked by cerumen or debris, then the effect of the force on tube displacement would be greater [4] (tympanostomy tube will migrate medially). In rare situations, the medial migration of the tympanostomy tube may also occur due to technical error [10], inattention or lack of experience. The optimal management of this condition has not been well delineated. It is established that symptomatic migration of the tympanostomy tube requires surgical removal [4, 7, 10]. In the literature, all cases who showed symptoms associated with the medial migration of the tympanostomy tube underwent surgery and had no postoperative complications [4, 8, 10] (Table 1). Some authors suggest that asymptomatic migrated tubes should be left alone with close follow-up of patients [7, 9, 10] (Table 1). Thus, the surgical removal of the medialized tube would only be performed once a symptom appears. We present an apprehension about this attitude, cause firstly, presence of foreign body in the middle ear cleft presents a potential risks of serious complications such as cholesteatoma and irreversible ossicular damages and secondly surgical removal of migrated tympanostomy tube has low morbidity, therefore we recommend a surgical removal for all medialized tubes even if the patient is asymptomatic. This surgery should be the least invasive possible. An approach via a myringotomy overlying the migrated tube, or through persistent perforation is preferred to a post-auricular approach. This posterior technic should be reserved for cases presenting a complication.

## Conclusion

In this report, we put the light on this rare complication of the tympanostomy tube emplacement often unknown by pediatricians and otolaryngologists. Once the medial migration of the tympanostomy tube is diagnosed and given the risk of serious sequelae that it can cause, we recommend surgical removal of the tube even in asymptomatic patients. We believe that myringotomy is the most optimal and safest management option. If contraindicated or facing a parental refusal of surgery, close follow-up should be instituted.

## Competing interests

The authors declare no competing interests.
